# Same Locus for Non-shattering Seed Pod in Two Independently Domesticated Legumes, *Vigna angularis* and *Vigna unguiculata*

**DOI:** 10.3389/fgene.2020.00748

**Published:** 2020-07-23

**Authors:** Yu Takahashi, Alisa Kongjaimun, Chiaki Muto, Yuki Kobayashi, Masahiko Kumagai, Hiroaki Sakai, Kazuhito Satou, Kuniko Teruya, Akino Shiroma, Makiko Shimoji, Takashi Hirano, Takehisa Isemura, Hiroki Saito, Akiko Baba-Kasai, Akito Kaga, Prakit Somta, Norihiko Tomooka, Ken Naito

**Affiliations:** ^1^Genetic Resources Center, National Agriculture and Food Research Organization (NARO), Tsukuba, Japan; ^2^Faculty of Animal Sciences and Agricultural Technology, Silpakorn University, Cha-Am, Thailand; ^3^Advanced Analysis Center, NARO, Tsukuba, Japan; ^4^Okinawa Institute of Advanced Sciences, Uruma, Japan; ^5^Department of Genome Medicine, National Center for Child Health and Development, Setagaya, Japan; ^6^Tropical Agriculture Research Front, Japan International Research Center for Agricultural Sciences, Ishigaki, Japan; ^7^Institute of Crop Science, NARO, Tsukuba, Japan; ^8^Department of Agronomy, Faculty of Agriculture at Kamphaeng Saen, Kasetsart University, Nakhon Pathom, Thailand; ^9^Center of Excellence on Agricultural Biotechnology (AG-BIO/PERDO-CHE), Bangkok, Thailand

**Keywords:** legume, azuki bean, yard-long bean, cowpea, secondary wall thickening, MYB26, pod shattering, domestication

## Abstract

Loss of pod shattering is one of the most important domestication-related traits in legume crops. The non-shattering phenotypes have been achieved either by disturbed formation of abscission layer between the valves, or by loss of helical tension in sclerenchyma of endocarp, that split open the pods to disperse the seeds. During domestication, azuki bean (*Vigna angularis*) and yard-long bean (*Vigna unguiculata* cv-gr. Sesquipedalis) have reduced or lost the sclerenchyma and thus the shattering behavior of seed pods. Here we performed fine-mapping with backcrossed populations and narrowed the candidate genomic region down to 4 kbp in azuki bean and 13 kbp in yard-long bean. Among the genes located in these regions, we found MYB26 genes encoded truncated proteins in azuki bean, yard-long bean, and even cowpea. As such, our findings indicate that independent domestication on the two legumes has selected the same locus for the same traits. We also argue that MYB26 could be a target gene for improving shattering phenotype in other legumes, such as soybean.

## Introduction

One of the most important phenotypic changes during crop domestication is loss of seed shattering (Doubley et al., 2006). In legumes, this has been achieved by loss of pod shattering *via* malformation of dehiscence zone (Dong et al., 2014) or loss of helical shape change in the pod (Suanum et al., 2016; Murgia et al., 2017). However, this trait still needs to be improved as shattering problems sometimes force farmers to lose more than 20% of their annual yield (Philbrook and Oplinger, 1989). Thus, elucidating genetic mechanisms of shattering is important to reduce harvest loss.

Among the angiosperms, legumes have evolved a unique and sophisticated manner of shattering (Fahn and Zohary, 1955). Legume seeds are enclosed in seed pods, which explosively split open with its two valves twisting helically away from each other upon maturity (Armon et al., 2011). This mechanism is much more complicated compared to cereals, where matured seeds freely fall by abscission layer developed in pedicels (stalk of individual flower) (reviewed by Dong and Wang, 2015). *Brassica* and *Arabidopsis* are more similar to legumes in that the seeds are embedded in the siliques that also spring open upon maturity (Spence et al., 1996). However, their siliques do not exhibit any helical shape change as legume pods do. As such, knowledge obtained from rice and *Arabidopsis* cannot be simply applied to understand the shattering of legumes.

The key of helical shape change of legume seed pods is the development of a thick sclerenchyma with a bilayer structure on the endocarp (inner surface of the pod). In this bilayer structure, cellulose microfibrils of the outer and inner layers run at ±45° from the longitudinal axis of seed pods (Erb et al., 2013). As the matured seed pods dry, the microfibrils shrink in perpendicular directions and generate the helical force to blow the seeds off (Erb et al., 2013). In contrast, the endocarp of *Arabidopsis*, which develops cell layers with thickened secondary walls, is inflexible (Spence et al., 1996). As the matured siliques dry, the pericarp tissues shrink but the endocarp cell layers do not, generating tension to spring open the siliques from the dehiscence zone at the valve margins (Spence et al., 1996).

However, no specific genes have been identified for the sclerenchyma formation, despite several quantitative trait loci (QTL) analyses and genome-wide association studies locating several loci involved in legumes’ pod shattering (Dong et al., 2014; Suanum et al., 2016; Murgia et al., 2017; Lo et al., 2018; Rau et al., 2019). In soybean, the domestication-type allele of *SHAT1-5* disturbs only dehiscence zone formation and does not affect helical shape change (Dong et al., 2014). Fiber content in seed pods, which is related to sclerenchyma formation, is reduced in common bean (Murgia et al., 2017; Parker et al., 2019; Rau et al., 2019), but the responsible gene is not cloned yet. An exception is *Pdh1* gene in soybean, which reduces the helical force of the sclerenchyma when disrupted (Funatsuki et al., 2014). However, the plants with non-functional PDH1 protein still initiate sclerenchyma formation, so PDH1 seems involved in later steps in sclerenchyma development.

Contrary to soybean, azuki bean [*Vigna angularis* (Willd.) Ohwi et Ohashi] and yard-long bean [*Vigna unguiculata* (L.) Walp. cv-gr. Sesquipedalis E. Westphal] seem disrupted in initiating sclerenchyma formation. In azuki bean, an important legume crop in East Asia, the seed pods of domesticated accessions normally form a dehiscence zone, but the pericarps are thinner and tenderer and show little helical shape change (Isemura et al., 2007; Kaga et al., 2008). In yard-long bean, which is often cultivated in Southeast Asia, pods are very long (60–100 cm) and do not show any helical shape change at all (Kongjaimun et al., 2012). In addition, the pods are extremely tender, and thus young pods are favored as vegetables (Kongjaimun et al., 2013; Suanum et al., 2016). Therefore, we consider azuki bean and yard-long bean are the best materials to isolate the gene for initiating sclerenchyma formation.

Thus, in this study, we performed fine-mapping to identify the responsible locus for pod shattering and pod tenderness in azuki bean and yard-long bean, respectively. Interestingly, we have previously revealed that the QTLs controlling pod shattering in azuki bean and pod tenderness in yard-long bean are co-localized around 8–10 cM on the linkage group 7 (LG7) (Kongjaimun et al., 2013), which corresponds to Chr7 in azuki bean (Sakai et al., 2015) and Chr5 in cowpea (Lonardi et al., 2019). To narrow down the candidate region, we developed backcrossed populations and DNA markers based on the genome sequence of azuki bean (Sakai et al., 2015), cowpea (Lonardi et al., 2019), and the wild cowpea (sequenced in this study). Our efforts identified MYB26 transcription factor as the only candidate.

## Materials and Methods

### Plant Materials and Growth Condition

**FIGURE 1:**
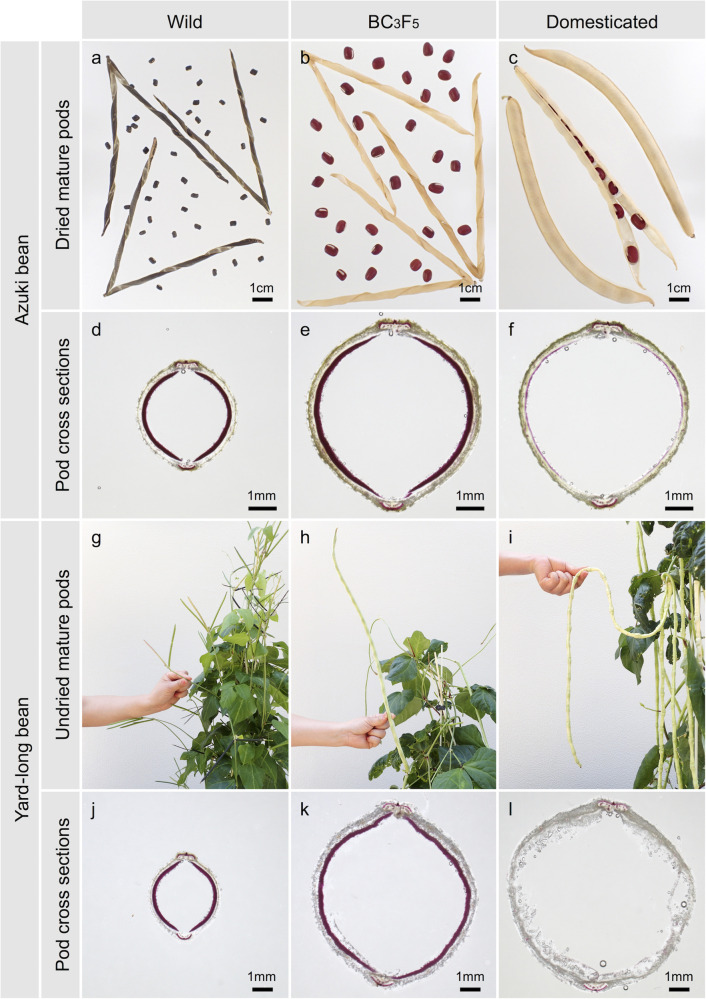
Pod phenotypes of parental accessions and BC_3_F_5_ plants with wild-type phenotypes in genetic background of domesticated genotypes. **(a,d)** Wild azuki bean (JP107881). **(b,e)** BC_3_F_5_ plant with shattering phenotype. **(c,f)** Domesticated azuki bean (JP81481). **(g,j)** Wild cowpea (JP81610). **(h,k)** BC_3_F_5_ plants with hard pod phenotype. **(i,l)** Yard-long bean (JP89093). **(a–c)** Dried mature pods. **(d–f,j–l)** Cross sections of pods stained with phloroglucinol. **(g–i)** Young pods.

All the accessions used in this study were provided by NARO Genebank (Tsukuba, Japan) (Figure 1 and Supplementary Figure 1).

For azuki bean, we started from the BC_1_F_2_ plants derived from a cross between domesticated azuki bean (JP81481, the recurrent parent) and a wild relative, *Vigna nepalensis* Tateishi & Maxted (JP107881) (Isemura et al., 2007). Of them, we selected one BC_1_F_2_ plant where the locus for pod shattering is fixed with the wild-type allele but most of other loci are fixed with domestication-type alleles and crossed again to the recurrent parent. We further backcrossed the obtained BC_2_F_1_ plants to obtain BC_3_F_1_ plants. We selfed them and obtained BC_3_F_2_ seeds from those with pod shattering phenotypes. For BC_3_F_3_, BC_3_F_4_, and BC_3_F_5_ populations, we kept selecting and selfing those with recombination within the candidate region. We also included plants that were heterozygous throughout the candidate region for obtaining more recombination and some that are homozygous in the same region to reconfirm the relationship of genotype and phenotype.

For yard-long bean, we started from BC_1_F_2_ plants derived from a cross between yard-long bean (JP89083, the recurrent parent) and a wild cowpea, *Vigna unguiculata* subsp. *dekindtiana* (Harms) Verdc. (JP81610) (Kongjaimun et al., 2013). Of them, we selected one BC_1_F_2_ plant that are fixed with wild-type allele at the locus for pod tenderness but are fixed with domestication-type at most other loci. The selected BC_1_F_2_ plants were backcrossed to the recurrent parent and further backcrossed to obtain BC_3_F_1_ plants. We selfed them and obtained BC_3_F_2_ seeds from those with hard pods phenotype. We kept selecting and selfing up to BC_3_F_5_ populations as done in azuki bean.

All the plants were grown from July through November in a field located in Tsukuba city, Japan, except BC_3_F_5_ plants of yard-long bean that were grown in a greenhouse from September through January.

### Observation of Sclerenchymal Tissue

We observed sclerenchymal tissues in seed pods by staining lignin with the Wiesner (phloroglucinol–HCl) reaction (Pormar et al., 2002). We harvested three undried mature seed pods per plant, sliced them to 200-μm sections with a Plant Microtome MTH-1 (Nippon Medical & Chemical Instruments Co., Ltd., Osaka, Japan), incubated in phloroglucinol–HCl solution for 1 min and observed with stereoscopic microscope SZX7 (OLYMPUS, Tokyo) for BC_3_F_5_ plants of azuki bean and yard-long bean. Phloroglucinol–HCl solution was prepared by dissolving 1 g of phloroglucin in 50 ml ethanol and adding 25 ml of concentrated hydrochloric acid.

### Phenotyping

For azuki bean populations, we evaluated pod shattering by counting the number of twists in a pod, as described in Isemura et al. (2007). For BC_3_F_2_ and BC_3_F_3_, we harvested five pods per plant before shattering and measured the length. We then incubated the pods at 60°C to let them totally dry. Unless the pods dehisced, we manually opened the pods and counted the number of twists and calculated the number of twists/cm. For BC_3_F_4_ and BC_3_F_5_, we harvested 10 pods per plant, let them dry, and evaluated the rate of shattering pods before counting the number of twists.

For yard-long bean, we manually evaluated the tenderness of five young pods per plant by binarizing hard or soft.

We also measured 100 seed weight on BC_3_F_2_ and BC_3_F_5_ plants of the azuki bean population and BC_3_F_4_ and BC_3_F_5_ plants of the yard-long bean population.

### Sequencing and Assembly of the Wild Cowpea Genome

We sequenced the whole genome of wild cowpea (JP81610) with RSII sequencer (Pacific Biosciences, Menlo Park, CA, United States), as we have done previously for azuki bean (Sakai et al., 2015). DNA was isolated from 1 g of unexpanded leaves with the cetyl trimethylammonium bromide (CTAB) method and purified with Genomic Tip 20/G (Qiagen K. K. Tokyo). The extracted DNA was sheared into 20-kb fragments using g-TUBE (Covaris, MA, United States) and converted into 20-kb SMRTbell template libraries. The library was size selected for a lower cutoff of 10 kb with BluePippin (Sage Science, MA, United States). Sequencing was performed on the PacBio RS II using P6 polymerase binding and C4 sequencing kits with 360 min acquisition. In total, 21 SMRT cells were used to obtain ∼26.4 Gb of raw reads.

In total, 4 million PacBio reads were used for *de novo* assembly with Canu v1.6 under the default settings (corOvlErrorRate = 0.2400, obtOvlErrorRate = 0.0450, utgOvlErrorRate = 0.0450, corErrorRate = 0.3000, obtErrorRate = 0.0450, utgErrorRate = 0.0450, cnsErrorRate = 0.0750). About 23.5x error corrected and trimmed reads longer than 1,000 bp were assembled to contigs. Assembled contigs were polished by PacBio raw reads by using the arrow in GenomicConsensus v2.3.2 (Pacific Biosciences of California, Inc.).

Repeat detection was conducted by RepeatMasker ver. 4.0 (Smit et al., 2013)^1^. A *de novo* repeats library of wild cowpea genome constructed by RepeatModeler ver. 1.0.11 (see footnote 1) and the Fabaceae repeats library in RepBase24.02 (Smit and Hubley, 2008)^2^ were used for the prediction.

*Ab initio* gene prediction was done with AUGUSTUS (version 3.3.2) (Stanke et al., 2008). A set of gene annotation information of recently published cowpea genome was used for training AUGUSTUS (Lonardi et al., 2019). We trained a new model twice using 1,000 high-confidential genes selected based on abundance of annotations of domains, pathway networks, and gene ontology information. BUSCO v3 (Waterhouse et al., 2017) was used to evaluate protein sequences of annotated genes.

Single-nucleotide polymorphisms (SNPs) and short insertions/deletions (indels) were detected by using MUMmer v3.23 (Kurtz et al., 2004). Genome alignment of our wild cowpea assembly and the reference cowpea genome (Lonardi et al., 2019) was conducted by the nucmer command with the following options: –maxgap = 500 –mincluster = 100. Then one-to-one alignment was extracted with delta-filter command with an option: −1. Based on the alignments, SNPs and indels were reported by the show-snps command.

Raw sequence data, the assembled sequences, and gene annotations are all available from DNA Data Bank of Japan (DDBJ)^3^ under the BioProject ID PRJDB8129.

### Genotyping

We extracted DNA from the seeds as described by Kamiya and Kiguchi (2003) and genotyped them by fragment analysis with capillary electrophoresis for simple sequence repeats (SSRs) and INDELs as described by Isemura et al. (2007) or by directly sequencing SNP sites (see section “Direct Sequencing” below). The information of the markers we used is summarized in Supplementary Table 1.

We designed primers by Primer3 (Untergasser et al., 2012) to amplify polymorphic sites between the domesticated azuki bean and *V. nepalensis* that are available in *Vigna* Genome Server (*Vig*GS^4^; Sakai et al., 2016) and those between the domesticated cowpea genome in Legume Information System^5^ and the wild cowpea genome sequenced in this study. Parameters for designing primers on Primer3^6^ were 20–30 bp in length, 55°C–65°C in annealing temperature, and 100–350 bp and >700 bp in expected length of amplified fragments for fragment analysis and direct sequencing, respectively.

For azuki bean, the markers we used were as follows:

BC_3_F_2_: CEDG064 and SPD01-SPD07
BC_3_F_3_: CEDG064, SPD03, SPD04, SPD08, SPD09
BC_3_F_4_: CEDG064 and SPD08–SPD11
BC_3_F_5_: CEDG064, SPD10, SPD11 and the sequencing primers for Vigan.07G034400

For yard-long bean, the markers we used were as follows:

BC_3_F_4_: VuPT01–VuPT06
BC_3_F_5_: VuPT03, VuPT04, VuPT07–VuPT13 and the sequencing primers for Vigun05g273300, Vigun05g273400, and Vigun05g0723500.

### Direct Sequencing

To sequence the (potentially) polymorphic sites, we amplified the template DNA with AmpliTaq Gold 360 Master Mix (Thermo Fisher Scientific K. K., Tokyo), performed sequencing reaction with BigDye Terminator v3.1 (Thermo Fisher Scientific K. K., Tokyo), and sequenced with ABI Genetic Analyzer 3130xl (Thermo Fisher Scientific K. K., Tokyo), according to the provider’s protocol.

### Determining Transcribed Sequences of Vigan.07g34400 and Vigun05g273500

To sequence both the domesticated and the wild alleles of Vigan.07g034400.01 and Vigun05g273500 genes, we sequenced the transcribed sequences of both loci. We extracted total RNA from 100 mg of flower buds right before flowering of all the parental accessions using RNeasy Plant Mini Kit (QIAGEN) with RNase-free DNase I (Invitrogen). Total RNA of 1 μg was converted into first-strand cDNA with Super Script II Reverse Transcriptase (Invitrogen) and Oligo(dT)12-18 Primer (Invitrogen) following the manufacturer’s instructions. The cDNA sequences of Vigan.07g034400.01 and Vigun05g273300.01 were then amplified and sequenced with the primers (Supplementary Table 1) and then transferred to the direct-sequencing protocol described above.

## Results

### Anatomical Analysis of Pod Sclerenchyma

To confirm that azuki bean and yard-long bean have lost or reduced sclerenchyma in seed pods, we observed cross sections of seed pods stained with phloroglucinol–HCl and found a clear-cut difference between the wild and domesticated accessions. In the wild azuki bean and cowpea, which have shattering phenotypes, thick layers of sclerenchyma (∼0.3 mm) were formed on the endocarps of seed pods (Figure 1). However, in the domesticated azuki bean and yard-long bean, which are both non-shattering, the sclerenchyma layer was slightly formed (<0.1 mm) or not formed at all, respectively (Figure 1).

We also evaluated the correlation between the thickness of pod sclerenchyma and helical shape change in seed pods (number of twists/cm) in the BC_3_F_5_ plants of azuki bean and yard-long bean. As a result, all the shattering plants in azuki bean population formed thicker sclerenchyma (0.15–0.20 mm) and showed a stronger helical shape change (0.30–0.43) than the non-shattering plants did (0.00–0.08 mm and 0.00–0.05, respectively) (Figure 1 and Supplementary Figure 1). The correlation coefficient between the sclerenchyma thickness and the helical shape change was 0.92. In the yard-long bean population, all the plants with hard pod phenotype formed sclerenchyma (0.16–0.22 mm) and showed little helical shape change (0.09–0.12), whereas those with soft pod phenotype did not form sclerenchyma or show helical shape change at all (Figure 1 and Supplementary Figure 1). The correlation coefficient between the sclerenchyma thickness and the helical shape change was 0.99.

In addition, we observed cross sections of seed pods in a wild soybean and three domesticated soybean cultivars and found thick sclerenchyma layers in all the accessions (Supplementary Figure 2).

### Fine-Mapping Pod Shattering in Azuki Bean

**FIGURE 2:**
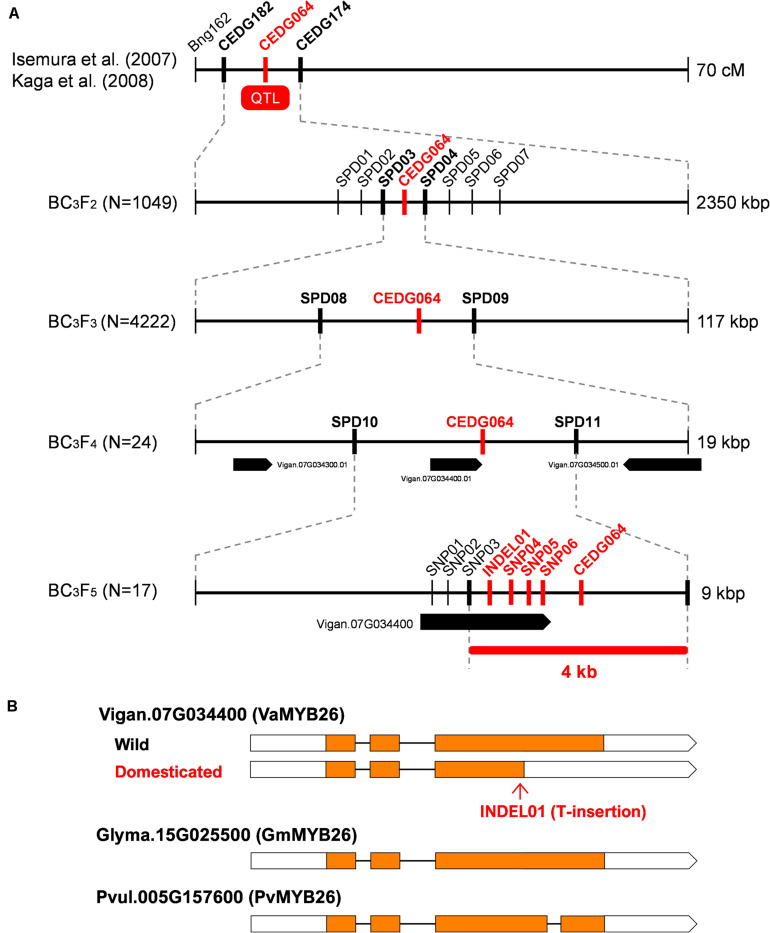
Fine-mapping of pod shattering factor in azuki bean. **(A)** Process of fine mapping. Vertical lines indicate DNA marker sites. Red markers indicate those with complete linkage with the phenotype in the mapping population. Bold black markers indicate those neighboring the candidate region. **(B)** A schematic of the wild-type and domesticated-type alleles of Vigan.07g034400. The MYB26 orthologues of soybean (Glyma.15G025500) and common bean (Pvul.005G157600) are also shown. Boxes indicate exons, whereas lines indicate introns. Coding DNA sequences (CDS) are filled with yellow.

We previously mapped the QTL for pod shattering in between CEDG182 and CEDG174 on LG7, which is around 1.4–4.0 Mbp in Chr7 of the reference azuki bean genome (Sakai et al., 2015) (Figure 2). To more finely locate the genetic factor for pod shattering, we genotyped 1,049 BC_3_F_2_ plants and selected 238 for phenotyping. The obtained phenotype and genotype data revealed the pod shattering factor was completely linked with CEDG064 and located in between SPD03 and SPD04 (Figure 2 and Supplementary Table 2). We selected 46 plants out of the 238 (Supplementary Table 2), selfed them, and obtained 4,222 BC_3_F_3_ seeds.

Of the BC_3_F_3_ seeds, we selected 53 that had recombination between SPD03 and SPD04. Phenotyping and genotyping on these 53 revealed that pod shattering factor was still completely linked with CEDG064 and was in between SPD08 and SPD09 (Figure 2 and Supplementary Table 3), which is about 19 kb long and contained three genes (Figure 2).

Since this region seemed to have a relatively higher recombination rate, we expected it would be possible to further locate the pod shattering factor. Thus, of the 53 BC_3_F_3_ plants, we selected 24 plants (Supplementary Table 3) and selfed them to obtain BC_3_F_4_ seeds. We then cultivated 1–7 plants/line (81 in total; Supplementary Table 4) for further genotyping and phenotyping (Figure 2).

As a result, we again found CEDG064 was completely linked with the phenotype, but we also obtained 29 recombinants between SPD10 and CED064 and five between CEDG064 and SPD11 (Supplementary Table 4). Thus, the pod shattering factor was located in a 9-kb region between SPD10 and SPD11, where there is only one gene Vigan.07g034400 (Figure 2), which encoded VaMYB26, an ortholog of AtMYB26.

To see if there were any loss-of-function mutations in VaMYB26 in azuki bean, we searched the polymorphism data between wild and domesticated azuki bean provided by *Vig*GS. The database showed that there were six SNPs (SNP01-SNP06) and one INDEL (INDEL01) within its open reading frame (ORF). Of these, all the SNPs were in the introns or in the 3′-untranslated region (UTR), but the INDEL was within the coding DNA sequence (CDS) (a T insertion 4 bp before the stop codon in the domesticated azuki bean). Since the wild azuki bean did not have this T insertion, the CDS could be 405 bp (125 aa) longer (Supplementary Figure 3). Interestingly, a BLAST search of MYB26 gene revealed that the longer version is widely conserved across plant taxa (Figure 2). Thus, the VaMYB26 in the domesticated azuki bean seemed to have an immature stop codon.

To confirm the polymorphisms identified in the database, we determined the genomic and the transcribed sequences of VaMYB26 locus of both parents and found all the polymorphisms in the ORF were true (Supplementary Figure 3). We also sequenced the 17 lines of BC_3_F_5_, which we obtained from the selected BC_3_F_4_ plants (those with fixed genotypes between SPD03 and SPD09; Supplementary Table 5), to further confirm the relationship between the genotypes and the phenotype. Of the 17, one had recombination between SNP03 and INDEL01, but none had recombination between INDEL01 and CEDG064 (Supplementary Table 5). Thus, the genotypes at INDEL01, SNP04–SNP06, and CEDG064 were completely linked with the pod shattering phenotype, locating the pod shattering factor within the 4-kb region between SNP03 and SPD11 (Figure 2 and Supplementary Table 5).

### Assembly and Annotation of Wild Cowpea Genome

Though the reference genome of cowpea had been released (Lonardi et al., 2019), it was yet not easy to obtain polymorphic markers because the genome sequence of the wild cowpea was not available. Thus, to facilitate our fine-mapping the factor of pod tenderness, we sequenced and assembled the genome of *V. unguiculata* subsp. *dekindtiana* (JP81610), which is a wild cowpea accession used to develop the mapping population of yard-long bean. We used PacBio RSII and obtained 4,413,480 raw reads with an average read length of 6.0 kbp and N50 length of 10.6 kbp, covering 44.9X of the estimated genome size (585.8 Mbp) (Lonardi et al., 2019) (Supplementary Figure 4 and Supplementary Table 6). The assembly produced 4,285 contigs covering 488.5 Mbp (83.4%) of the cowpea genome, with the contig NG50 of 438.6 kbp and the maximum contig length of 2.6 Mbp, respectively (Supplementary Table 7). Of the assembled sequences, 45.7% composed of transposable elements, of which LTR retrotransposons share the largest content (20.5% of the assembly) (Supplementary Table 8). Our *ab initio* gene prediction detected 36,061 protein-coding genes, and 27,345 of those were non-repeat related (Supplementary Table 9). The annotated genes of the wild cowpea contained 93.2% (85.6% complete and 7.6% partial) of the 1,440 plant genes in BUSCO v3 (Waterhouse et al., 2017).

We aligned the genome sequences of the wild cowpea to those of the reference cowpea (Lonardi et al., 2019) and identified 5,661,319 SNPs and 1,626,169 INDELs. As expected, we detected fewer SNPS and INDELs around centromeric and pericentromeric regions but many in chromosome arms, which were presumably gene-rich regions (Supplementary Figure 5).

### Fine-Mapping Pod Tenderness Factor in Yard-Long Bean

**FIGURE 3:**
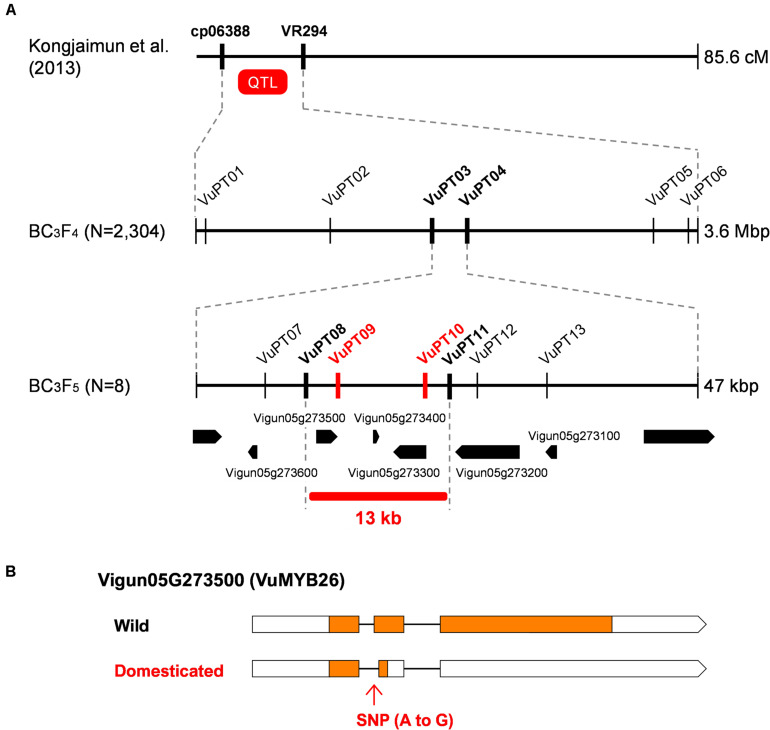
Fine-mapping of pod tenderness factor in yard-long bean. **(A)** Process of fine-mapping. Vertical lines indicate DNA marker sites. Red markers indicate those with complete linkage with the phenotype in the mapping population. Bold black markers indicate those neighboring the candidate region. **(B)** A schematic of the wild-type and domesticated-type alleles of Vigun05g273500. Boxes indicate exons, whereas lines indicate introns. Coding DNA sequences (CDS) are filled with yellow.

The QTL for pod tenderness was previously located between cp06388 and VR294 on LG7 (Kongjaimun et al., 2013) (Figure 3), which corresponds to about 47.5–45.5 Mbp region in Chr5 of the cowpea genome (*Vigna unguiculata* v1.0, NSF, UCR, USAID, DOE-JGI)^7^. To more finely locate the pod tenderness factor, we designed more markers based on the SNPs and INDELs we identified above, genotyped 2,304 BC_3_F_4_ seeds, and selected 195 for phenotyping (Supplementary Table 10).

The obtained data of genotype and phenotype located the pod tenderness factor between VuPT03 and VuPT04, which was about 47 kbp (Figure 3 and Supplementary Table 10). Although no marker was completely linked with the phenotype, eight plants had recombination between this region (Figure 3 and Supplementary Table 10).

Of the 195 BC_3_F_4_ plants we tested, we selfed the eight plants and obtained BC_3_F_5_ for further mapping. As a result, we located the candidate regions in between VuPT08 and VuPT11, which was 13 kbp long and contained three genes, Vigun05g27300, Vigun05g273400, and Vigun05g273500. Of note, Vigun05g273500 encoded VuMYB26 (Figure 3 and Supplementary Table 11).

According to the SNPs and INDELs data, Vigun05g273500 had an A to G substitution which might disrupt the junction site of the first intron and the second exon (Supplementary Figure 6). On the other hand, Vigun05g27300 seemed to have only synonymous SNPs, and Vigun05g273400 was a non-coding gene. All the SNPs were confirmed by directly sequencing the ORFs of these genes. Interestingly, the ORF sequences of the three genes were exactly the same between the reference cowpea and yard-long bean, which means yard-long bean and the reference cowpea share the domestication-type allele in this locus (Supplementary Figure 6).

To test whether the A to G substitution disrupted splicing, we sequenced cDNAs of this locus prepared from both parents. As a result, we found, in the domestication-type allele, the junction site of the first intron and the second exon had shifted downstream by 8 bp, which could result in a frameshift and an immature stop codon in the middle of the second exon (Supplementary Figure 6). This product would encode a protein of 60 aa, which would be 305 aa shorter than that of the wild-type allele.

### Seed Size Increase by Loss of Sclerenchymal Tissue

Because Murgia et al. (2017) suggested the pod shattering phenotypes load an energy cost on plants and limit seed size, we measured 100 seed weight of the mapping population (BC_3_F_2_ of azuki bean and BC_3_F_4_ of yard-long bean).

**FIGURE 4:**
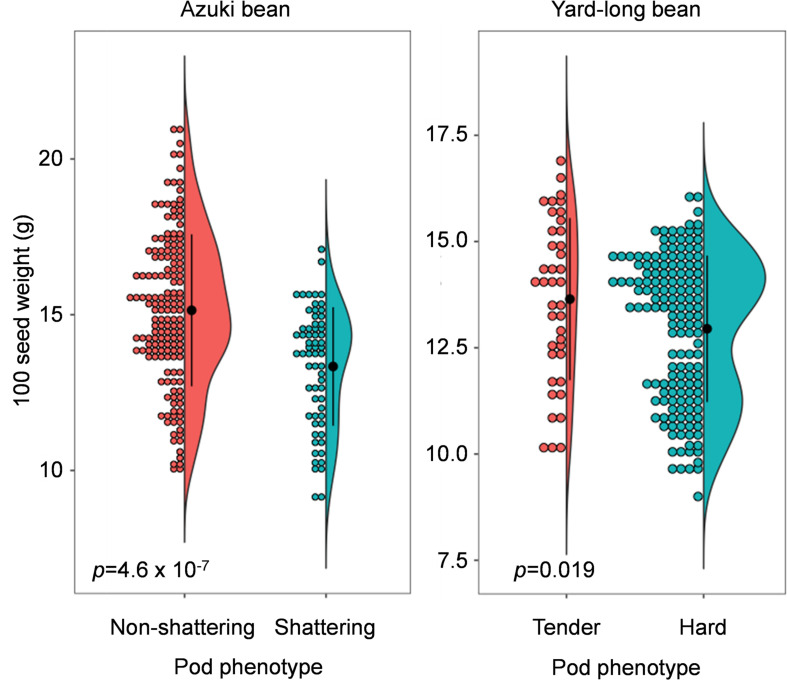
Dot-violin plot of 100 seed weight in azuki bean and yard-long bean mapping population. The bin of dot plots is 0.2 g. The black dots and vertical lines in the violin plots indicate the averages and standard deviations, respectively. The *p*-values of the *t*-test are also indicated in the plots.

As expected, those with domestication-type trait produced larger seeds compared to those with the wild-type trait (Figure 4). In the azuki bean population, the seeds of non-shattering plants were 15.4 ± 2.4 g/100 seeds, whereas the seeds of shattering plants were 13.3 ± 1.9 g/100 seeds. In the yard-long bean population, the seeds of tender-pod plants were 13.6 ± 1.9 g/100 seeds, whereas the seeds of hard-pod plants were 12.9 ± 1.7 g/100 seeds. The following *t*-test revealed that in both cases, the seeds of the plants with the domestication-type phenotypes produced significantly larger seeds than the others (*p* = 4.6 × 10^–7^ for azuki bean and *p* = 0.019 for yard-long bean).

We also measured 100 seed weight of BC_3_F_5_ plants and observed the same trend, though the differences were not significant (Supplementary Figure 7). In azuki bean, the seeds of non-shattering plants were 9.7 ± 1.4 g/100 seeds, whereas the seeds of shattering plants were 8.7 ± 1.2 g/100 seeds. In yard-long bean, the seeds of tender-pod plants were 14.2 ± 1.5 g/100 seeds, whereas the seeds of hard-pod plants were 12.2 ± 1.2 g/100 seeds.

## Discussion

In this study, we identified Vigan.07g034400 (Figure 2) and Vigun05g273500 (Figure 3) are the best candidate genes for sclerenchyma formation in the seed pods of azuki bean and yard-long bean, respectively (Figure 1). The domestication-type alleles of both loci encode truncated proteins due to immature stop codons (Supplementary Figures 3, 6), leading to a reduction or loss of sclerenchyma, which is required to generate the helical tension for splitting open legume pods (Figure 1 and Supplementary Figure 1). Although our primary goal of the fine-mapping in yard-long bean was to identify a gene for the extreme tenderness of its seed pod, the SNP we identified was in common between yard-long bean and the reference cowpea (Supplementary Figure 6). Thus, the domestication-type allele of Vigun05g273500 is not the specific allele to yard-long bean but is the one which has originally been selected for resistance to pod shattering during domestication of cowpea (Lush and Evans, 1981).

Vigan.07g034400 and Vigun05g273500 are orthologous to each other and to *Arabidopsis* transcription factor MYB26, which is strongly expressed in the anther and promotes secondary wall thickening in the endothelium (Yang et al., 2007). Interestingly, this secondary wall thickening is critical for anther dehiscence and thus for releasing the matured pollen (Yang et al., 2007). However, this gene is hardly expressed in siliques and is not involved in pod dehiscence (Yang et al., 2007). On the other hand, MYB26 is expressed in seed pods in many legume species according to the expression database [*Vig*GS, the *Phaseolus vulgaris* Gene Expression Atlas (*Pv*GEA)^8^ and Soybean eFP Browser^9^ ]. In addition, Di Vittori et al. (2020) has recently revealed in common bean that reduced expression of MYB26 is a key for non-shattering genotypes. Thus, in legumes, MYB26 plays a crucial role in the development of pod sclerenchyma and has been a common target of human selection among the three independently domesticated legumes, azuki bean, cowpea, and common bean. This fact suggests the importance of MYB26 in pod shattering, despite there are usually multiple options to achieve a domestication-related trait (Doubley et al., 2006; Takahashi et al., 2019).

Our results also indicate that the accumulation of some more mutations is necessary to achieve the extreme tenderness of seed pod in yard-long bean. Indeed, the QTL analysis on pod tenderness (Kongjaimun et al., 2013) and pod fiber content (Suanum et al., 2016) identified a few other QTLs than the locus we identified here. Thus, such mutations had been selected for the complete loss of pod sclerenchyma during the secondary domestication in Asia (Faris, 1965).

In addition, we have to admit that availability of whole-genome sequences greatly facilitated the study. Although the lower coverage and shorter read length of our assembly resulted in ∼10 times lower contiguity than the reference cowpea genome (Lonardi et al., 2019), its accuracy was good enough as all the SNPs between the two assemblies, which we tried to use as markers, were confirmed to be true (Supplementary Tables 5, 11 and Supplementary Figures 3, 6). We also reemphasize the power of map-base cloning, which is a classic, time-consuming, but reliable approach to isolate responsible genes.

We also consider the knowledge obtained in this study is applicable to improve soybean or other legume crops. As described before, soybean has reduced shattering ability by disturbing dehiscence zone formation between the valves (Dong et al., 2014) and by reducing helical tension of the sclerenchyma (Funatsuki et al., 2014). However, it still forms thick sclerenchyma in the seed pods (Supplementary Figure 2), which causes shattering problems especially under drought conditions (Philbrook and Oplinger, 1989). Thus, loss-of-function mutations or genome-editing in MYB26 genes may reduce sclerenchyma and enforce resistance to shattering. In addition, our findings indicate that loss or reduction of sclerenchyma may result in a slight (∼10%) increase in seed size (Figure 4 and Supplementary Figure 7). Murgia et al. (2017) also reported that common bean plants with non-shattering genotypes produce slightly larger seeds than those with shattering genotypes. This might be because developing sclerenchyma loads an energy cost for plants that could be allocated for seed production (Murgia et al., 2017), or pod hardness limits space for seed enlargement. In any case, MYB26 may serve not only for reducing harvest loss but also for directly increasing seed size.

## Conclusion

Our map-based cloning approach with support of whole-genome sequences identified MYB26 as an only candidate for the development of pod sclerenchyma, which generates helical tension of legumes’ shattering pods. Our findings suggest that MYB26 might be a good target to improve resistance to pod shattering in soybean and other legumes.

## Data Availability Statement

The datasets presented in this study can be found in online repositories. The names of the repository/repositories and accession number(s) can be found in the article/ Supplementary Material.

## Author Contributions

YT, PS, AKo, and KN planned the study. YT, AKo, CM, YK, TI, HSai, AKa, NT, and KN performed the experiments and collected the data. MK, HSak, KS, KT, AS, MS, and TH performed the genome sequencing, *de novo* assembly, and gene annotation. YT, AKa, HSak, MK, and KN analyzed the data. KN wrote the manuscript. All the authors contributed to the article and approved the submitted version.

## Conflict of Interest

TH is currently employed by the company Sentan Pharma Inc., his contribution to this study was made while he was working for his former affiliation, OIAS.

The remaining authors declare that the research was conducted in the absence of any commercial or financial relationships that could be construed as a potential conflict of interest.

## Footnotes

1 http://repeatmasker.org

2 https://www.girinst.org

3 https://www.ddbj.nig.ac.jp/index.html

4 https://viggs.dna.affrc.go.jp

5 https://legumeinfo.org/

6 http://primer3.ut.ee

7 http://phytozome.jgi.doe.gov/

8 http://plantgrn.noble.org/PvGEA/index.jsp

9 http://bar.utoronto.ca/efpsoybean/cgi-bin/efpWeb.cgi
